# Altered **p**16^**INK**4^ and RB1 Expressions Are Associated with Poor Prognosis in Patients with Nonsmall Cell Lung Cancer

**DOI:** 10.1155/2012/957437

**Published:** 2012-04-30

**Authors:** Weiqiang Zhao, Cheng C. Huang, Gregory A. Otterson, Marino E. Leon, Yan Tang, Konstantin Shilo, Miguel A. Villalona

**Affiliations:** ^1^Department of Pathology, The Ohio State University Medical Center, Columbus, OH 43210, USA; ^2^The Division of Oncology, Department of Medicine, The Ohio State University Medical Center, Columbus, OH 43210, USA; ^3^Department of Anatomic Pathology, H. Lee Moffitt Cancer Center, Tampa, FL 33612, USA

## Abstract

p16^INK4^ and RB1 are two potent cell cycle regulators to control the G1/S transition by interacting with CDK4/6, E2F, and D-type cyclins, respectively. Depending on the tumour type, genetic alterations resulting in the functional inactivation have frequently been reported in both genes. By contrast, much less is known regarding the overexpression of these proteins in the tumor cells. In this study, expressions of p16^INK4^ RB1, and *CDKN2A* copy number variances (CNV) in the tumor cells were assessed by immunohistochemistry and fluorescence in situ hybridization (FISH), respectively, in 73 nonsmall cell lung cancer (NSCLC) with known 5-year survivals. The histologic type (*P* = 0.01), p16^INK4^ (*P* = 0.004), and RB1 (*P* < 0.001) were predictive of survivals. The *CDKN2A* CNV (*P* < 0.05) was also significant when compared to those cases without CNV. Therefore, among the molecular genetic prognostic factors, expressions of RB1 and p16^INK4^ in the tumor cells were the most strongly predictive of adverse outcomes in stage I and II nonsquamous NSCLC.

## 1. Introduction

Primary lung carcinoma is one of the leading causes of cancer death worldwide. Genetic and molecular alterations involving tumorigenesis have been extensively studied. Inactivation of tumor suppressor genes by deletion, mutations, altered splicing, promoter mutations, or epigenetic modifications are the common causes in lung cancers [1–3]. Amplification and activation mutations of oncogenes are often account for many malignant behaviors and worse clinical outcomes [4, 5]. In fact, most of these genetic alterations might directly or indirectly affect the cell cycle and proliferation of the tumor cells. p16^INK4^ and RB1 are two important tumor suppressor proteins and participate in negatively regulating the proliferation of normal cells [6–8]. Like other tumors, studies were focused on the genetic alterations resulting in either loss or decreased expressions and functions in the tumor cells because of their inhibitory roles in cell proliferation [9–14]. By contrast, studies were limited regarding the overexpression of these proteins and their effects on the tumorigenesis and prognosis in the tumor cells. Reports become more prominent in the head and neck squamous carcinomas in which p16^INK4^was overexpressed under the viral effect by the high-risk serotypes of the human papilloma virus (HPV), though sparse reports in tumors like basal-like breast carcinoma and NSCLC [15–17]. A single study showed that the combined RB-negative/p16-positive/cyclin D1-negative tumors in NSCLC might relate to the adverse outcomes, but the independent role of each proteins (p16^INK4^ and RB1) in the unfavorable prognosis was not confirmed [17]. In this paper, we studied p16^INK4^ and RB1 protein expressions and *CDKN2A* gene copy variances in NSCLC with special reference to an association of the abnormal individual protein expression with clinical characters.

## 2. Materials and Methods

### 2.1. Case Selections and Tissue Microarray

A tissue microarray (TMA) was prepared from formalin-fixed paraffin-embedded (FFPE) tissue specimens from 1985 to 1997 acquired through the pathology archive services of the Ohio State University Medical Center, Columbus, OH, USA. All the cases selected for this study meet following criteria: (1) nonsquamous NSCLC, surgically managed patients with stage I or stage II NSCLC at the time of diagnosis; (2) available clinical followup and outcome data; (3) adequate tissue (all surgical resection specimen) for immunohistochemical stains (IHC) or molecular studies. Patients selected for this study received no neoadjuvant chemotherapy or radiotherapy prior to surgery. Seventy-three NSCLC cases met the criteria and were included in this study. All the cases were reviewed, and the pathology diagnosis of each case was reclassified according to the current WHO classification. The study has been approved by the institutional human research committee. Additionally, tissues from human brain, lung, lymph node, kidney, placenta, thyroid, heart, liver, testes, and adrenal glands (1-2 samples each) were included in the TMA as normal controls.

### 2.2. Immunohistochemistry (IHC)

Immunohistochemistry was done using monoclonal p16 antibody clone INK4 (MTM laboratories) or pRB clone 13A10 (NovoCastra Laboratories) on a DAKO-automated staining instrument (Dako Scientific Systems, Tucson, AZ, USA) using an ABC-based detection kit (I View DAB, Ventana Medical Systems) or polymer-based detection kit (Mach3, Biocare Medical) as described previously [18, 19]. Staining intensity was scored semiquantitatively separately for the cytoplasm and/or nucleus, using a scale from 0 to 3: 0, no staining; 1+, weak intensity in more than 25% of nuclei; 2+ moderate and 3+, strongly positive intensity in more than 75% of nuclei. Tumor cells with moderate (2+) or strong (3+) stainings were graded as overexpression or positive, while none (0) and weak (1+) stainings were negative. Specimens were scored in a blinded fashion by two pathologists (W. Zhao and M. E. Leon).

### 2.3. Interphase Fluorescence In Situ Hybridization (FISH)

To investigate the *CDKN2A* gene copy number variances (CNV), a dual color chromosome 9 centromere, *CEP9* (spectrum green), and *CDKN2A* gene spectrum (orange) probe kit were used (Vysis, Abbott Laboratories, Abbott Park, IL) on the paraffin-embedded tissues (FFPE), either on the TMA or full sections at 2 to 4-*μ*m-thickness as described previously with modifications [19]. Normally, each nucleus was expected to have 2 copies of each *CEP9* (reference numbers of chromosome 9) and *CDKN2A* gene, that is, a cell without CNV should have ratio of 1 (2 *CDKN2A*/2 *CEP9*). The loss of *CDKN2A* might be homozygous (0/2, ratio = 0) or heterozygous (1/2, ratio = 0.5). The gains of *CDKN2A* might be amplification (>4/2, ratio >2.1) or polysomy 9 (both *CDKN2A* and *CEP9* were amplified). All of the images and FISH slides were reviewed by a pathologist (W. Zhao) using a fluorescence microscope (Olympus BX51), and images were taken with a digital image camera (DP70, Olympus, USA).

### 2.4. In Situ Hybridization of HPV High- and Low-Risk Probes

The sample was processed and detected for HPV-H and -L risk probes as described previously [20]. 

### 2.5. Survival and Statistical Analysis

Patient survival status at five years (60 months) after initial pathologic diagnosis was obtained from the institutional information warehouse at The Ohio State University and used as the outcome variable to calculate the five-year overall survival rate. Univariable analyses were performed using Chi-square (*χ* ^2^) test or Fisher's exact test to study the associations of categorical variables to the outcomes. *P* value of <0.05 was considered statistically significant. Statistical analyses were performed using MedCalc software (Frank Schoonjans, Mariakerke, Belgium).

## 3. Results

A total 73 cases of nonsquamous NSCLC at stage I and II were enrolled in this study. Overall, 32 out of 73 (43.8%) patients survived more than 5 years after the diagnosis. The factors with no correlation to the outcomes were age (median 65.0 years, 34–85) and gender (males: 57.5% versus females: 42.5%).

Histologically, there were 51 adenocarcinoma, 9 large cell carcinoma, 8 large cell neuroendocrine carcinoma, and 5 mucoepidermoid tumors, and they were grouped into two categories, adenocarcinomas (51, 69.9%), and nonadenocarcinoma (22, 30.1%). The patients with adenocarcinoma lived longer than those with nonadenocarcinomas (52.9% versus 22.7%, *P* = 0.01).

**Figure 1:**
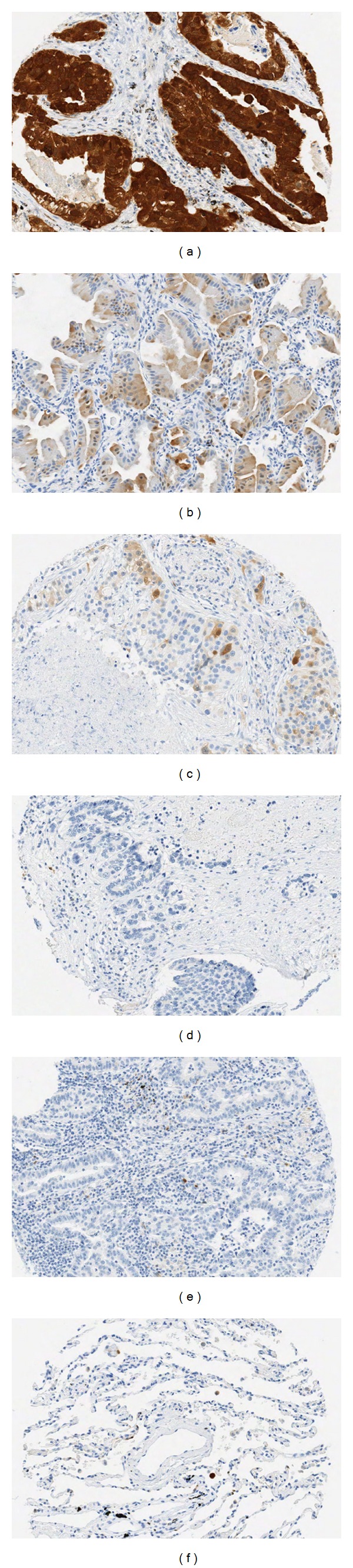
p16^INK4^ immunohistochemical staining in lung cancer samples. The patterns in tumor cells range from strong (3+) in an adenocarcinoma with acinar differentiation, moderate (2+) in a mixed adenocarcinoma, and weak (1+) in a mixed adenocarcinoma from (a) to (c), respectively. No (0) expression was seen in tumor cells from a large cell neuroendocrine carcinoma (d) and an adenocarcinoma with acinar differentiation (e-f). There is no expression in normal lung tissue (f) or stromal including normal lymphocytes of the the tumor samples (a–e).

Immunohistochemical stains for p16^INK4^ and RB1 were performed on all samples (*n* = 73). The prevalence of p16^INK4^-positivity was 30.1% (22/73). The representative IHC results were shown in Figure 1. It was present both in cell nucleus and cytoplasm, and ranged from strong to moderate (Figures 1(a) and 1(b)). The negative cases (69.9%) included both 1+ (*n* = 6) (Figure 1(c)) and 0 (*n* = 45) (Figures 1(d) and 1(e)) expressions. The proteins were not detected in the normal lung tissues (Figure 1(f)) or the tumor stromas (Figures 1(a)–1(c)). The p16^INK4^-positive rates between adenocarcinomas and nonadenocarcinoma were not statistically significant (31.9% versus 26.9%, *P* > 0.05).

The presence of p16^INK4^ in the tumor cells was associated with unfavorable outcomes (RR: 1.8142, 95% CI: 1.2642–2.6037, *P* = 0.004) (Table 1). Among the survived subjects, the positive rate was 12.5% (4/32). In contract, among the dead subjects, it was 43.9% (18/41), a 3.5-fold higher than the survived group. Although being adenocarcinoma was a favorable factor, cases with p16^INK4^-positive adenocarcinomas were significantly predictive of shorter survivals (RR: 2.0, 95% CI: 1.155–3.4623, *P* = 0.035) than the negative ones (Table 1). Their relationship in nonadenocarcinoma tumors was not done due to the low numbers.

**Figure 2:**
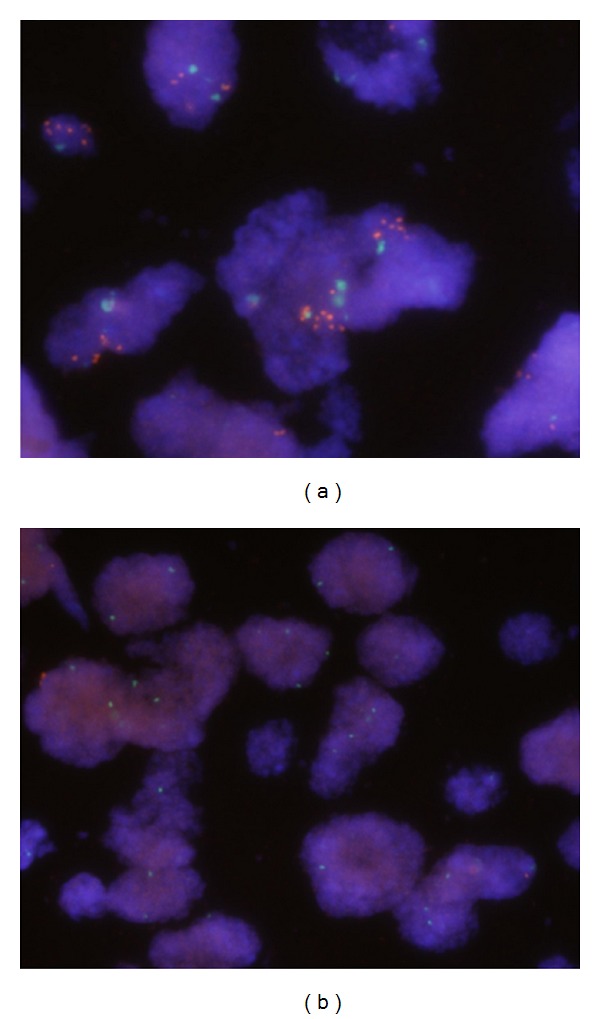
Gene copy variances of *CDKN2A* evaluated by FISH. (a) Representative example of tumor cells with increased CDKN2A gene copy numbers: the multiple red *CDKN2A* signals with fewer green *CEP9* signals indicate the amplification (ratio >2.1). (b). Representative example of tumor cells with homozygous loss: the two or multiple green *CEP9* signals but absent of red *CDKN2A* signals in tumor cells indicate specific loss of this gene rather than total loss of chromosome 9 (ratio = 0). Note a possible stromal cell with a normal signal pattern (2 red and 2 green) at the lower right corner of (b).

A relationship between p16^INK4^ IHC and CNV of *CDKN2A* assessed by FISH was studied.  Figure 2 illustrated representative cases with amplification (Figure 2(a)) and homozygous loss (Figure 2(b)), respectively. Of 73 cases tested, the prevalence of CNV was 31.5% (23/73) which included both gains (3 amplification and 6 polysomy) and losses (5 homozygous loss and 9 heterozygous) (Table 1). The IHC results were obviously proportional to the gains (7/9, 77.8%) or homozygous losses (0%, 0/5) of *CDKN2A* gene (Table 2). In cases with heterozygous loss, 6 of 9 (66.7%) were positive for IHC. In contrast, 18% (9/50) samples without CNV had positive p16^INK4^ which reflects uncertainty of the association of IHC and CNV in these two groups. As a result, only 31.8% (7/22) p16^INK4^-positive tumors were caused by the gain of *CDKN2A* gene.

*CDKN2A* abnormalities were more often seen in nonadenocarcinoma than adenocarcinoma, but the difference was not statistically significant (40.9% versus 27.5%) (*P* > 0.05). When compared to those with normal CDKN2A gene copies, the presence of CNV in *CDKN2A* in the tumors was associated with the unfavorable outcomes (RR: 1.5399; 95% CI: 1.056–2.245; *P* < 0.05) (Table 2).

In the head and neck carcinoma, p16^INK4^ positivity was positively associated with the infection of the high-risk human papilloma viruses (HPV) [20]. We examined this correlation by in situ hybridization (ISH) on the 73 tumor samples, and none (0/73, 0%) was positively detected with HPV-H and HPV-L probes (Data not shown).

**Figure 3:**
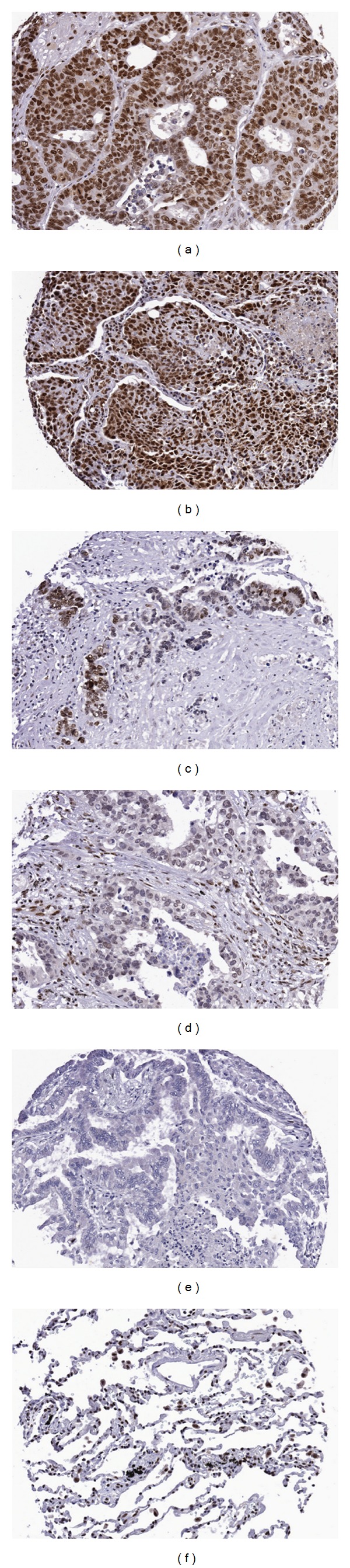
RB1 immunohistochemical staining in lung cancer samples. The represesntative patterns of expression range from strong (3+) in a mixed adenocarcinoma (a), a large cell neuroendocrine carcinoma (b), moderate (2+) in a large cell neuroendocrine carcinoma (c), and weak (1+) in an adenocarcinoma with acinar differentiation, respectively, in the nuclei of tumor cells. No (0) expression was seen in tumor cells of an mixed adenocarcinoma (e). However, weak expression (1+) is present in normal lung tissue (f) or stroma.

RB1 expression was readily detected in the nuclei by IHC ranging from strong (3+ to 2+), weak (1+), to absent, as illustrated in Figure 3. In contrast to p16^INK4^, expression of RB1 (1+) was detected in the nuclei of the normal control tissues from heart, lung, thyroid, testes, adrenal gland, prostate, and kidney but variably in the stromal endothelial, fibroblast, and lymphoid cells. The expression of low level of RB1 in the normal tissues might be due to the fact that RB1 promoter are reminiscent which might be associated with housekeeping genes and result in the ubiquitous expression of the RB1 gene [21].

Of the 73 cases, 41 were negative (0-1+, 56.2%), 32 were positive (2 to 3+, 43.8%) for RB1 expression, respectively (Table 3). The RB1-positive tumors were significantly associated with adverse outcomes (RR: 2.002; 95% CI: 1.309–3.06; *P* < 0.001) (Table 3), which were independent from other factors such as age, sex, histological subtypes, and p16^INK4^ expression. In adenocarcinoma or p16^INK4^-negative tumors, the positive RB1 expression was also associated with the unfavorable outcomes (RR = 2.833, 95% CI: 1.532–5.239; *P* < 0.001; and RR = 3.273, 95% CI: 1.632–6.562, *P* < 0.001, resp.). Some RB1-negative tumors in which no trace of RB1 was detected (15%, 11/73) had unfavorable outcomes. Their correlations to the outcomes were not statistically significant due to lack of enough samples (data not shown). Patients with both p16+/RB1+ tumors were all dead (100%, 8/8) in 5 years, which was much higher than p16+/RB1− (64%, 9/14) tumors, but a statistically significant correlation was not sought due to the low number of cases.

## 4. Discussion

In this study, we demonstrated that factors associated with poor outcomes in stage I and II nonsquamous NSCLC included nonadenocarcinoma, positive expression of p16^INK4^ and RB1 by IHC, and with CNV of *CDKN2A* gene in the tumor cells.

Consistent with previous reports, p16^INK4^ was undetectable in the normal tissues or the stromal cells of the tumor tissues. The absent expression of p16^INK4^ in the tumor cells, however, might be caused by homozygous loss of *CDKN2A* gene as demonstrated in this study or hypermethylation of the *CDKN2* promoters as seen in other tumor types [22, 23]. The causes of the overexpression in the tumor cells might resulted from genetic abnormalities, viral effect or a tumor-associated mutant of *CDKN2A* [24–26]. We demonstrated that in NSCLC, 32% of the p16^INK4^-positive cases was resulted from the increased copy numbers of *CDKN2A*. The HPV viral effect was ruled out by negative ISH results in this study. Therefore, studies for the mechanisms that result in an upregulated p16^INK4^ expression should be sought in the future.

The overexpression of p16^INK4^ protein in tumor cells is not uncommon findings. For example, p16^INK4^ was increased in multiple ovarian cancer cell lines as well as in 7 of 10 clinical ovarian cancer specimens [27]. Previous reports also showed that p16^INK4^-positive prostate cancers were associated with early relapse and relapse, its association to an unfavorable prognosis in NSCLC is not known yet [28–30]. The paradoxical p16^INK4^ positivity in NSCLC associated to the poor outcome is demonstrated in this study though interpretation needs to be cautious due to relatively small numbers included.

Like p16^INK4^, loss of RB1 function by genetic deletion is commonly seen to be an essential process of oncogenesis in wide ranges of human malignancy, such as retinoblastoma, breast cancer, and small cell carcinoma of the lungs. The increased RB1 in tumor cells was puzzling but was observed in colorectal carcinoma and bladder tumors [31, 32]. In this study, we demonstrated that RB1 positivity in NSCLC was often seen (43.8%) in NSCLC. Furthermore, we demonstrated that its overexpression was significantly associated with the adverse outcomes. In addition, in those p16^INK4^-negative or adenocarcinoma tumors, the RB1 status stratifies them into favorable and unfavorable groups.

Abundant functionally defective mutant protein might be produced in the tumor cells, but their significance to the clinical outcomes is not clear yet [33, 34]. The significance of increased expression of functionally intact tumor suppressor proteins such as p16 and RB1 in malignant cells remains poorly understood but might be explained by the concept of the cellular homeostasis in the cancer cells. For example, the apoptosis that normally resulted from Myc overproduction can be suppressed in tumor cells by oncogenic mutations that stimulate survival signals or directly inhibit the apoptotic machinery. Therefore, in order to couple with the hyperproliferation stress, the tumor cells might increase the production of cell-cycle inhibitory proteins such as p16INK4 to suppress the G1/S transition. The tumor cells with low proliferation might be more resistant to radiation and chemotherapy. Furthermore, the roles of overexpressed RB1 in suppressing the apoptosis might result in resistance to therapeutic radiation or chemotherapy, too [35, 36].

In conclusion, we demonstrated that tumors with higher expression of p16^INK4^ and RB1 were statistically significantly associated with unfavorable outcomes in patients with stage I and II nonsquamous NSCLC. The stratification of these patients by profiling p16^INK4^and RB1 protein expression in the tumors might provide predictive biomarkers for cancer prognosis. Further works to understand how these tumor suppressor genes were abnormally upregulated, and their roles in cancer homeostasis are needed to provide scientific bases for the prevention interference. 

## Figures and Tables

**Table 1:** Comparison of p16, histology, FISH, and five-year survival rate^1^.

p16 IHC	Overall*		Adenocarcinoma**		Nonadenocarcinoma***		CNV CDKN2A***			
	Alive	Dead	Alive	Dead	Alive	Dead	Gain (ratio > 2)	Loss (ratio = 0)	Loss (ratio = 0.5)	Normal (ratio = 1)
Positive	4 (12.5)	18 (43.9)	5 (18.5)	12 (50.0)	0 (0.0)	5 (29.4)	7 (77.8)	0 (0.0)	3 (33.3)	12 (10.0)
Negative	28 (87.5)	23 (56.1)	22 (81.5)	12 (50.0)	5 (100.0)	12 (70.6)	2 (22.2)	5 (100)	6 (66.7)	35 (90.0)

^1^The data presented in the table in format of “case number (% of the same column)”.

Fisher's probability exact test (two tailed): *P = 0.004, **P = 0.036, ^∗∗∗^not done.

**Table 2:** Correlation of *CDKN2A* copy number variances with the outcome and the tumor types^1^.

*CDKN2A* CPN	Survival*		Histologic type**	
	Alive	Dead	Adenocarcinoma	Nonadenocarcinoma
CNV	6 (18.8)*	17 (41.5)	14 (27.5)	9 (40.9)
Normal	26 (81.2)	24 (58.5)	37 (72.7)	13 (49.1)

^1^The data presented in the table in format of “case number (% of the same column)”.

Fisher's probability exact test (two tailed): *P = 0.0455 and **P = 0.282.

**Table 3:** Results of RB IHC scores and five-year survival rate^1^.

RB score	Overall*		Adenocarcinoma**		Nonadenocarcinoma***		p16-negative*	
	Alive	Dead	Alive	Dead	Alive	Dead	Alive	Dead
Positive	7 (21.9)	25 (61)	5 (18.5)	15 (62.5)	3 (42.8)	10 (58.8)	6 (20.0)	17 (70.8)
Negative	25 (78.1)	16 (39.0)	25 (81.5)	9 (37.5)	4 (57.1)	7 (41.2)	24 (80.0)	7 (29.2)

^1^The data presented in the table in format of “case number (% of the same column)”.

Fisher's probability exact test (two tailed): *P < 0.001, **P < 0.05; ^∗∗∗^not done.
